# Manufacturability is access: redesigning antibody production for equitable pediatric access in LMICs

**DOI:** 10.3389/fped.2026.1810896

**Published:** 2026-05-18

**Authors:** Kerry R. Love, Stacy E. Martin, Laura Crowell Orella

**Affiliations:** Sunflower Therapeutics PBC, Medford, MA, United States

**Keywords:** alternative hosts, continuous manufacturing, in-country manufacturing, manufacturability, microfactory

## 1 Introduction

Monoclonal antibodies (mAbs) have become an essential tool for pediatric health, with innovative products now available for infectious disease prophylaxis in infants (1) and expanded approvals of critical treatments for use in children with serious rare diseases and disorders (2). Access to these life-saving treatments is severely limited for most patient populations globally, however, due to high costs of manufacturing and inconsistent registration protocols with global regulatory agencies (3, 4). Patients in the US, Canada and Europe account for 80% of the mAb market, with only about 15% of the global population receiving these products (3). Current manufacturing practices for mAbs result in a Cost of Goods Manufactured (COGsM) of ∼US$50–100/g, severely restricting access in Low- and Middle-Income Countries (LMICs) where price sensitivity is high, particularly for treating children. Significant efforts have been undertaken to achieve a target COGsM of <US$10/g through optimizing existing processes by intensification and application of continuous manufacturing approaches, but even optimized processes still hit limits of >US$30/g (5). Further cost reductions will require prioritizing mAb product “manufacturability”—designing improved products with simpler manufacturing and engineering host biology to enable straightforward manufacturing easily deployed in automated facilities. Facility and process automation significantly reduce the cost of labor (6) and help to reduce barriers to clinical manufacturing in LMICs, creating a pull for partnership between the regional workforce and regulatory authorities. By establishing regional production, LMICs can be transformed from “price takers” who must accept prices set by global manufacturers into “market makers” with a competitive landscape that benefits patients (7), especially pediatrics.

## 2 High-impact pediatric mAb products and accessibility

Monoclonal antibodies continue to transform medicine, through their use as highly effective treatments and diagnostic tools. The use and applications of mAbs continues to evolve, including major breakthroughs like using mAbs as proactive prophylaxis for specific diseases in infants and young children. Long acting mAbs against *P. falciparum* infection, such as CIS43LS, MAM01, and L9LS, can address a critical gap in malaria prevention in children when the administration of multi-dose vaccines and preventative chemotherapies is limited by eligibility, use, and coverage challenges (8–11). In a recent Phase 2 trial in Mali, L9LS protected children aged 6–10 years over a 6-month malaria season against *P. falciparum* infection and clinical malaria (10, 11). The use of mAbs to treat children for global health concerns like malaria could be a critical gateway to the broader use of mAbs in LMICs, since the dose requirements, and therefore costs, are substantially lower than for adult patients and could render them more likely to be viewed as cost effective overall.

Another powerful clinical success of mAbs in pediatric patients is the use of Beyfortus™ (nirsevimab) during the respiratory syncytial virus (RSV) season for the prevention of RSV lower respiratory tract disease (LRTD) in newborns and infants. While RSV remains the leading cause of infant hospitalization in the US, hospitalization rates have declined dramatically since the introduction of RSV prevention products such as maternal vaccination and nirsevimab (12, 13). The recent approval of Enflonsia™ (clesrovimab) in several countries, and ongoing global regulatory filings, should further expand the availability of RSV prophylactic mAbs for the protection of newborns and infants worldwide. Even though the costs to use mAbs as an intervention for pediatric patients are generally higher than those for vaccines, current data indicates that mAbs ultimately are more cost-effective due to superior health outcomes (14–16). We acknowledge, though, that efforts should be undertaken to investigate the potential suitability and preference for maternal vaccines over administration of mAbs to infants in various scenarios, including different geographies, to best understand the value of different prophylactic strategies worldwide. In British Columbia, a model deploying a universal nirsevimab program for all infants resulted in a massive reduction in RSV-related healthcare burden, but its estimated incremental cost-effectiveness ratio (ICER) of CAD$271,651 per quality-adjusted life year (QALY) makes it economically unjustified at current prices. Even in a high-income country like Canada, the unit price would need to drop from CAD$450 to approximately CAD$110 per dose for a universal mAb strategy to become preferred (16). Similarly, an examination of the potential impact and cost-effectiveness of RSV prevention strategies across 131 LMICs showed mAbs to be more cost effective than maternal vaccines, but only at similar product prices of US$3–5 per dose (14). Over 97% of all RSV-attributable deaths globally in children under 5 occur in LMICs (17). As of 2026 nirsevimab was not approved for clinical use in any country in Africa, but regulatory filings recently have been made in eight LMICs elsewhere, including Argentina, Brasil, China, India, Malaysia, Paraguay, Thailand and Türkiye (18).

While the current price of nirsevimab (approx. US$495/dose) renders it a non-starter for most African health budgets, cost is not the sole barrier. The product's design—optimized for temperate 'seasonal' RSV—clashes with the potential need for multiple doses in countries with longer RSV seasons, compounding costs. Furthermore, the reliance on fragile cold-chain infrastructure for importation and storage limits mAbs' current reach to urban centers. True access requires not just a cheaper product, but a regionally adaptable one—manufactured locally to minimize logistics and potentially re-engineered for greater stability or potency against local viral variants (19). These efforts must be further supported by innovative licensing arrangements, robust technology transfers and streamlined regulatory and policy solutions (20). Together, reductions in price and improved local availability can ensure that mAbs like nirsevimab are deployed in LMICs, providing the performance data in the context of local disease variants and co-morbidities (malaria, HIV, malnutrition), critical to justify regional health ministries spending their limited budgets.

## 3 How can the base price of mAbs be dramatically reduced? Key levers of COGsM

While there are many contributors to the price of consumer goods generally, including manufacturing costs, quality control, distribution, and value-based profit margins, the base price of mAbs, or any biopharmaceutical product, is effectively set by the COGsM, which is a combination of direct costs for drug substance and drug product manufacturing, like amortized capital expenditures, raw materials and process/product consumables, and indirect costs related to facility operation, like labor, utilities and analytics. Multiple cost contributors, including major determinants of COGsM, will need to be lowered to decrease the base price of mAbs (21). Continuous bioprocessing methods can enable substantial cost reductions (>20%) in drug substance production as compared to legacy batch production (6) by integrating distinct unit operations, eliminating hold times and significantly reducing human interventions through process connectivity and automation (22). This transformative approach offers numerous additional benefits, including smaller upfront capital costs, improved product quality, reduced waste, and enhanced manufacturing agility and sustainability. Labor excluded, continuous bioprocessing can reduce COGsM for mAbs to US$35/g, albeit for productivities of >5 g/L/day and annual throughputs of >>100 kg (5). When combined with the use of single-use technologies, continuous bioprocessing enables maximal facility utilization through multi-product manufacturing (23), which can further offset facility-related indirect costs dependent on product demands.

For mAbs, key direct cost drivers are related to the complex manufacturing platforms used to make them, whether in batch or continuous bioprocesses (Table 1) (3, 5, 6, 21). Classical methods rely on mammalian cell culture (e.g., CHO cells) and affinity chromatography with Protein A for product purification, which are inherently expensive processes (24). Beyond resins for purification, the general cost of raw materials (cultivation media, buffers, other purification substrates) required for these complex processes contributes to the high COGsM. Achieving the COGsM target of <US$10/g will require innovating an end-to-end continuous bioprocess capable of delivering productivity well over 5 g/L/d space-time yield (STY) using minimal cultivation media (<US$5/L) and simple purification materials (<US$5/g). Recently, eukaryotic microbes and protozoa*,* including *Pichia pastoris*, *Thermothelomyces heterothallica* C1 and *Leishmania tarentolae,* have demonstrated their capability as cost effective production hosts for the secreted expression of complex biomolecules, including mAbs with humanized glycosylation (25). While some of these organisms have less clinical experience, they possess characteristics ideal for achieving ultra-low COGsM, such as fast growth to ultra-high biomasses required to achieve the required STY even using simple nutrition sources. Accomplishing routinely high STYs with these organisms will likely require both host engineering for improved protein expression (26) and adaptation of product candidate amino acid sequences and post-translational modifications for a given host, like removal of known proteolytic sites (27), acceptance of truncated humanized glycoforms (GlcNAc-Man5 in Leishmania) (28) or substitution of post-translational modifications with protein-based functional elements capable of delivering desired clinical benefits (29). While adapting mAb candidates to be amenable for expression in simpler hosts, developers could optimize product sequences for other desirable clinical and biophysical attributes, like circulation longevity (30) and thermostability (31), that would render them easier to deliver in low-resource environments.

**Table 1:** Comparison of current and future states of pediatric mAb access for infectious disease prophylaxis and treatment based on key cost drivers and manufacturing approach. Outcomes are forward-looking, based on implementing the future state drivers of COGsM.

System Attribute	Current State: Limited access	Future State: Significant access
Drivers of COGsM for mAbs		
Expression host	CHO cells	Alternative hosts (e.g., microbes, protozoa)
Purification method	Protein-A resin	Non-affinity methods without resins
Bioprocess integration	Batch bioprocessing	Continuous bioprocessing
Facility type	Large facilities (>1,000L)	Microfactories
Labor requirements	>>100 operators	Fully automated
Manufacturing location	Single-point manufacturing	Distributed regional manufacturing
Product complexity	Highly complex	Manufacturable-by-design
Outcomes		
COGsM	US$50–100/g	<US$10/g
Price	∼US$500 per dose	∼US$1–5 per treatment season
Logistics	Extensive cold chain	Reduced cold chain; Thermostable by design
Product Focus	Single products designed for blanket use with focus on Global North patients	Diverse products relevant to local patients and local health priorities

Bioprocesses using alternative hosts also benefit from simple purification strategies, as they secrete fewer host-cell proteins when expressing heterologous products, they are not contaminated by adventitious viruses, and they do not express endotoxins (25). Proteins expressed in microbial cultivation supernatants often have >80% initial purity, offering a unique opportunity to move away from conventional resins and columns to innovative scalable continuous purification methods like precipitation-filtration (32) or Aqueous Two-Phase Systems capable of liquid-liquid extraction using inert polymers (33–35). In the case that affinity methods are still required, convective Protein A membranes have recently achieved dynamic binding capacities >50 g/L with residence times of <15 s (vs. minutes for columns) (36). Each of these methods could support high yields of quality products with fast processing times in small facility footprints, all while using significantly cheaper raw materials compared to legacy approaches.

Unfortunately, even when using highly efficient alternative hosts in continuous processes, the indirect costs associated with mAb manufacturing in the Global North are unlikely to achieve COGsM below ∼US$20/g. To further lower COGsM, mAb manufacturing needs to go “lights out” (i.e., –fully automated with minimal to no human intervention) and be deployable wherever labor, land and existing facilities are most cost-effective, including rural areas and retrofitted brownfield spaces. Small, tractable, automated manufacturing units that can go anywhere and be operated by anyone have become the basis for microfactories, which are already achieving economies of scale worldwide for numerous consumer goods, like batteries, electric vehicles, home appliances and textiles (37). This approach is bringing manufacturing closer to consumers, providing risk mitigation for fragile supply chains and distribution networks, and delivering a profitable high mix of products at substantially lower volumes than legacy approaches. Correspondingly, simpler mAb products manufactured with inexpensive continuous processes could be deployed in fully automated microfactories using miniaturized equipment with completely disposable product contact surfaces (38). These facilities deploying drug manufacturing “in a box” would reduce the barriers to regionalized biotherapy production through low upfront capital needs and low ongoing operating costs, including lessening the skill demands of operators and highly classified space. Based on our cost modeling of bioprocessing technologies currently in development, the deployment of mAb manufacturing in LMICs using a microfactory approach is the only means to routinely achieve COGsM of <US$10/g for 1,000 kg or less annual demand of mAbs (Table 1).

## 4 How could regional production with microfactories further impact mAb accessibility?

Product accessibility is a combination of price and availability. While COGsM represent the ultimate minimum price, they are often only a fraction of the commercial price of a drug (10%–15% or less) as numerous other factors, including regional market dynamics, also contribute significantly to price (21). For example, markets with multiple domestic biologic drug manufacturers pay considerably less for products based largely on the competitive landscape (7). Local production potentially also increases the physical availability of products for regional health, as supply chains are simplified and manufacturers can be incentivized by governments or NGOs to manufacture for national health initiatives or specific population needs. The introduction of mAbs for rabies prophylaxis (Rabishield and subsequently Twinrab™) into the Indian market by Indian manufacturers (Serum Institute of India and Zydus Cadila) is a great example of in-country-for-country innovation and production solving a local health crisis (39). Local production could also reduce cold-chain requirements for certain products, reducing logistics and costs, though mAb product design for stability should also be prioritized. Availability for drugs, however, also depends on a functional regulatory environment with the capability and capacity for efficient review of product dossiers. In-country infrastructure for mAb production creates a local pull for national regulatory authorities (NRAs) to engage with manufacturers and see innovative manufacturing and analytical technologies in action benefitting review timelines and harmonization efforts essential for broadening drug access (4). A strengthened partnership between NRAs and manufacturers in LMICs can enhance appetites for innovation and clinical development, contributing to ecosystem building and creating market momentum with key stakeholders that results in additional products. Beyond strengthening regional manufacturing and regulatory capabilities, expanding therapeutic access will require growth in other aspects of the biopharma ecosystem in LMICs, including QA/QC, clinical development and product lifecycle management.

## 5 Discussion

True equity in pediatric mAb access cannot be achieved solely through charitable donations or tiered pricing from centralized hubs. It requires fundamental changes to the business models and pricing structures that currently determine how patients access mAb products (40). Specifically, the introduction of regional competition through multiple quality-focused domestic manufacturers producing mAbs designed for the needs of local patients could be game-changing for patient access in LMICs. Currently, healthcare systems in LMICs are beholden to monopolistic supply chains that dictate prices based on Global North overheads and value-based models. By deploying automated, simplified manufacturing platforms in-region, we unlock a new economic reality: cost-competition among local businesses based on local advantages with elimination of key markups, like international logistics premiums and import tariffs. Regional manufacturers in LMICs benefit from significantly lower operating expenses (OpEx)—specifically in labor and facility overheads—which are primary cost drivers in regulated biologics production. When local producers can manufacture high-quality antibodies at a structural cost advantage (targeting <US$10/g) (3, 21), they can adopt a local pricing structure that forces global suppliers to compete on price, driving the market floor downward. Furthermore, regional production in LMICs fosters health sovereignty, breaking the crippling financial and social dependence on external markets that creates massive healthcare-related trade deficits while leaving children in the Global South vulnerable to supply chain shocks. Regional production based on mAb manufacturability in microfactories is the prerequisite for a competitive, resilient global market that prioritizes patient access.
